# Frequency of Surgery in Black Patients with Malignant Pleural Mesothelioma

**DOI:** 10.1155/2015/282145

**Published:** 2015-04-30

**Authors:** Emanuela Taioli, Andrea S. Wolf, Jacqueline M. Moline, Marlene Camacho-Rivera, Raja M. Flores

**Affiliations:** ^1^Department of Population Health, Hofstra North Shore-LIJ School of Medicine, Great Neck, NY 11021, USA; ^2^Department of Thoracic Surgery, Mount Sinai Medical Center, New York City, NY 10029, USA

## Abstract

*Introduction*. Malignant Pleural Mesothelioma (MPM) is a rare disease, even less frequently described in minority patients. We used a large population-based dataset to study the role of race in MPM presentation, treatment, and survival. *Methods*. All cases of pathologically proven MPM were identified in the Surveillance, Epidemiology, and End Results (SEER) database. Age, sex, diagnosis year, stage, cancer-directed surgery, radiation, and vital status were analyzed according to self-reported race (black or white). *Results*. There were 13,046 white and 688 black MPM patients (incidence: 1.1 per 100,000 whites; 0.5 per 100,000 blacks; age-adjusted, *p* = 0.01). Black patients were more likely to be female, younger, and with advanced stage and less likely to undergo cancer-directed surgery than whites, after adjustment by stage. On multivariable analysis, younger age and having surgery were associated with longer survival for both cohorts; female gender (HR 0.82 (0.77–0.88)) and early stage at diagnosis (HR 0.83 (0.76–0.90)) were predictive of longer survival in white, but not in black, patients. *Conclusions*. Surgery was associated with improved survival for both black and white MPM patients. However, black patients were less likely to undergo cancer-directed surgery. Increased surgical intervention in MPM black patients with early stage disease may improve their survival.

## 1. Introduction

Malignant Pleural Mesothelioma (MPM) is an aggressive form of asbestos-related cancer with poor prognosis [1]. Possible treatments include surgery, chemotherapy, and radiation or a combination of them, but the effectiveness of these approaches is still debated. A randomized controlled trial conducted in the UK to assess the effectiveness of extra pleural pneumonectomy in terms of survival, complications, and quality of life [2] suggested that surgery did not offer any survival advantage in comparison to chemotherapy alone. To date, however, no US study has evaluated MPM incidence, treatment, and outcomes in African American patients. There are two recently published studies on MPM outcome based on SEER data. One series [3] reported survival in relation to histology and treatment but did not stratify or otherwise evaluate the data by race; the other analysis [4], conducted on a small sample of 294 black MPM patients, suggested that a smaller proportion of black patients received cancer-directed surgery, while the overall survival was comparable to that of white patients. This finding suggests that predictors of survival differ for black and white patients [4] and that the ethnic discrepancy in surgery rates may reflect a complex interaction between patients and/or surgeon choices, as well as clinical factors at diagnosis.

The present analysis of the SEER database was conducted to assess the incidence and temporal trends of MPM in a large population-based sample of black patients, to determine the rates of cancer-directed surgery and radiotherapy and if choice of treatment influenced survival in this population.

## 2. Material and Methods

The SEER database was explored from 1973 to 2009 to identify all cases of pathologically proven MPM within the site recode ICD-O-3 variable by ICD-O-3 morphology.

Cases who were diagnosed at age below 18 years, postmortem cases, nonmicroscopically confirmed cases, any living case without survival time in the database, and cases of malignant mesothelioma of other sites (retroperitoneal, peritoneal, genital, heart, mediastinum, soft tissue, digestive, other, and unknown primary site) were excluded. The SEER 09 registry includes data from 9 US registries, from 1973 to 2004; the SEER 17 registry includes cases from 17 registries, from 2000 to 2004; the SEER 18 registry includes cases from greater Georgia from 2000 onward, with the exception of adjustments for the areas impacted by Hurricanes Katrina and Rita.

### 2.1. Definition of Staging

Definition of staging is as follows:* localized*: invasive tumor confined to pleura; ipsilateral parietal and/or visceral pleura; mesothelioma with nodules beneath the visceral pleural surface; and localized, not otherwise specified;* regional*: extension to adjacent organs/structure: adjacent connective tissue, pericardium, endothoracic fascia, and diaphragm; visceral pleural invasion into lung parenchyma, lung involvement not otherwise specified; extension to adjacent organs such as the chest wall, ribs, myocardium, mediastinal organs, and tissues; mesothelioma with malignant pleural fluid/effusion; regional ipsilateral lymph nodes; and regional not otherwise specified;* distant*: contralateral pleura and lung, extension to intra-abdominal organs, cervical tissues, peritoneum, and metastasis; further contiguous extension; unknown if extension or metastasis; and distant lymph nodes.

### 2.2. Definition of Cancer-Directed Surgery

For cases diagnosed after 1998, patients were identified as having received cancer-directed surgery if any of the following codes were present for the “Rx Summ-Surg Prim Site” variable: 30 = simple/partial surgical removal of primary site; 40 = total surgical removal of primary site, enucleation; 50 = “debulking”; 60 = radical surgery which included partial or total removal of the primary site in continuity (partial or total removal) with other organs.

For cases prior to 1998, patients were identified as having received cancer-directed surgery if any of the following codes were present for the “Site Specific Surgery” variable: 10 = local surgical excision or destruction of lesion; 20 = partial/wedge/segmental resection; 30, 40 = lobectomy/bilobectomy with/without dissection of lymph nodes; 50 = complete/total/standard pneumonectomy, pneumonectomy, NOS; 60 = radical pneumonectomy plus dissection of mediastinal lymph nodes; 70 = extended radical pneumonectomy with diaphragm plus lymph nodes; 80 = surgery of regional and/or distant site(s)/node(s) only (includes removal of mediastinal mass only); 90 = resection of lung, not otherwise specified; surgery, not otherwise specified.

For all cases, code 00 (which indicated “no surgical procedure had been performed”) and the codes for other types of surgery (codes 01, 02, 03, 04, and 05) were used to categorize patients who did not undergo cancer-directed surgery.

### 2.3. Statistical Analysis

Age, sex, diagnosis year, stage, cancer-directed surgery, radiation, and vital status were analyzed (chemotherapy data not available) according to self-reported race (black and white). Comparisons between black and white patients were performed using the *t*-test for continuous variables and chi-square for categorical variables. Overall survival was defined as the time between initial date of diagnosis and either date of death or date of last follow up, whichever came first. Comparison of survival between blacks and whites was performed using multivariable regression methods based on a Cox proportional hazards model. All analyses were performed using SAS version 9.2 (Cary, NC, USA).

## 3. Results

**Figure 1:**
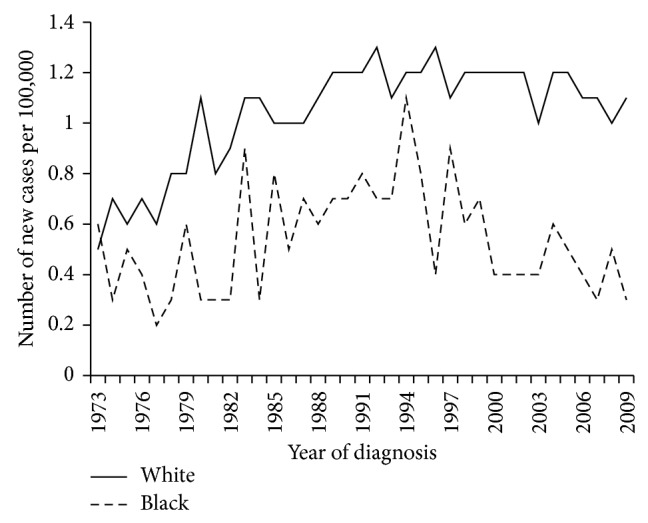
Age-adjusted incidence rates for blacks and whites, 1973–2009 (note: cases from SEER 9 database are included).

MPM age-adjusted incidence rates were 1.1 per 100,000 in whites and 0.5 per 100,000 in blacks (*p* = 0.01). The significant difference in rates has been present since 1973, when the data recording started (Figure 1). There were 13,046 white and 688 black patients with pathologic diagnosis of MPM (Table 1). Black patients were more likely to be female (26% versus 22%, *p* = 0.01) and younger (67 versus 70, *p* < 0.0001) and to present with more advanced stage of disease (65% versus 59%, *p* = 0.002).

### 3.1. Therapy

Black patients were less likely to receive cancer-directed surgery than white patients (18% versus 24%, *p* = 0.001). Black patients were more likely than whites to undergo no surgery or radiation (73% versus 68% received no treatment; *p* = 0.005). There was an inverse trend in undergoing surgery with more advanced stage in both ethnicities: among whites, 31.5% of localized cases received surgery, versus 21.4% of the advanced stage white patients. Among black patients, the corresponding proportions were 29.3% for localized disease and 15.1% for advanced stages (Table 3).

### 3.2. Survival

**Figure 2:**
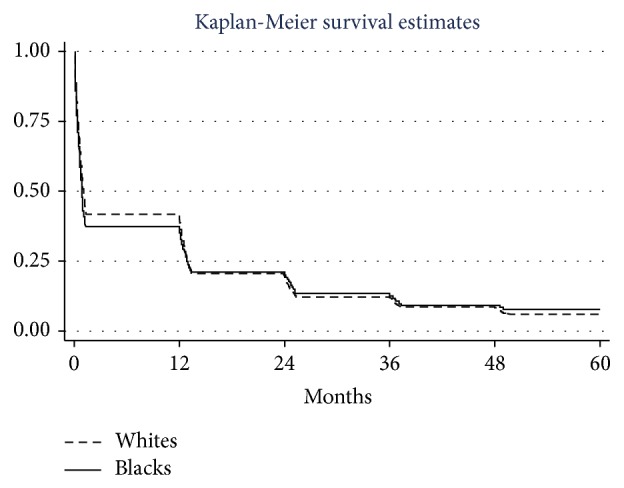
MPM survival according to ethnicity, 1973–2009. Log-rank chi-square: 0.45; *p* value 0.5012; Wilcoxon test: 4.13; *p* value 0.042.

Overall survival was similar for black and white patients (Figure 2). On multivariable analysis, female gender, younger age, early stage, and cancer-directed surgery were independent predictors of longer survival in white patients; younger age and surgery were associated with longer survival in black patients (Table 2). Three- and 5-year survival for patients who did not receive treatment was 7.9% and 3.5%, respectively, in whites and 10.6% and 6.7% in blacks.

## 4. Discussion

This SEER analysis includes the largest sample of black patients with MPM to date and demonstrates that MPM incidence is significantly lower in blacks compared to whites. Reasons for this large difference may reside in the lower opportunity for occupational asbestos exposure among blacks. The U.S. Department of Labor periodically publishes summary statistics on the US workforce [5] and reports that black workers are highly represented in the health and education services, public administration, and transportation, while they are less likely to be employed in those occupations where asbestos has been used in the past, such as building and ship construction, insulation, and mining. In addition, fewer blacks served in the U.S. Navy, which was historically a source of asbestos exposure for many men [6, 7]. The observation that there is a higher proportion of women among black (compared to white) MPM patients is a new finding and warrants further investigation. Blacks are also diagnosed with MPM at an earlier age than whites, which could reflect earlier exposure to asbestos than whites or alternatively may reflect more aggressive disease with earlier clinical symptoms.

Black MPM patients are diagnosed at a later stage than white patients; this may be due to general delays in seeking care for medical symptoms, lack of access to diagnostic procedures, lack of timely diagnosis when symptoms occur, lack of access to appropriate medical care, and/or lack of availability of insurance coverage. Another possible factor could be that symptoms at MPM onset are different in black compared to white patients, as previously reported in lung cancer [8], and this could cause delay in diagnosis. The role of each of these factors cannot be elucidated in this study, as the information is not available in the SEER database.

When the therapeutic approach is analyzed according to ethnicity, black patients underwent less cancer-directed surgery than white patients, as suggested by previous analysis of a smaller SEER dataset [4]. Similar to the observed lower rates of cancer-directed surgery in black MPM patients, a study conducted in early stage non-small cell lung cancer [9] demonstrated a low rate of surgical treatment among blacks. Black MPM patients were less likely to undergo either surgery or radiation in comparison to white patients. Although late stage at diagnosis was thought to be the main reason for this difference in treatment with regard to race, the stratified analysis by stage does not support that hypothesis, as blacks underwent less cancer-directed surgery than whites at every stage of MPM. Reasons for less surgical treatment could be the presence of comorbidities in black patients at the time of MPM diagnosis or the fact that patients were diagnosed in community-based hospitals lacking adequate surgical expertise or that surgery was not considered to be a treatment option. A recently published paper [10] indicates that race disparities exist in access to high-volume hospitals, a proxy for better quality of care in complex cases [11], even after adjustment for socioeconomic and insurance status.

When performed, cancer-directed surgery was associated with higher survival in both ethnic groups. Unfortunately, we were not able to analyze what type of surgical approach was performed (extrapleural pneumonectomy or radical pleurectomy/decortication) or if the approach differed according to ethnicity. Another limitation of the SEER dataset is that it does not contain information on any chemotherapy regimen administered to the patients. Other limitations include the broad definition of stage and the limited quality of the histology classification. Differences in these variables may have contributed to the observed health care disparities in rates of cancer-directed surgery, for example, if a higher proportion of blacks had nonepithelial (less favorable cell-type) disease compared to whites. However, the use of the SEER database allowed us to conduct the largest study, to our knowledge, of MPM black patients.

This analysis indicates that black patients with MPM are treated less frequently with surgery than their white counterparts, at every stage of their disease, but they experience similar survival as white MPM patients. This suggests that further attention to the treatment of black patients with MPM is needed to determine if black MPM patient survival would be improved with better access to the same treatments as whites. Further studies on exposure history, access to care, type of medical and surgical treatment, and hospital characteristics in black patients with MPM are needed.

## Figures and Tables

**Table 1:** Patient, disease, and treatment characteristics among whites and blacks (*n* = 13734).

Variable	Categories	Whites (*n* = 13046)	Blacks (*n* = 688)	*p* value
Sex	Male	10159 (78%)	507 (74%)	0.010
	Female	2887 (22%)	181 (26%)	

Mean age in year (95% CI)		70.2 (70.0–70.4)	66.6 (65.6–67.6)	<0.0001

Overall stage	Localized	1449 (11%)	75 (11%)	0.002
	Regional	2155 (16%)	91 (13%)	
	Distant	7639 (59%)	450 (65%)	
	Unknown	1803 (14%)	72 (11%)	

Cancer-directed surgery	No	9959 (76%)	563 (82%)	0.001
	Yes	3087 (24%)	125 (18%)	

Radiation	No	11387 (87%)	611 (89%)	0.241
	Yes	1659 (13%)	77 (11%)	

Therapy	No	8933 (68%)	505 (73%)	0.005
	Surgery only	2454 (19%)	106 (15%)	
	Radiation only	1026 (8%)	58 (9%)	
	Both surgery and radiation	633 (5%)	19 (3%)	

Mean survival in months (95% CI)		15.5 (15.1–15.9)	16.7 (14.5–19.0)	0.899

SEER, Surveillance, Epidemiology, and End Results.

**Table 2:** Effect of patient, disease, and treatment characteristics on 5-year survival (*n* = 13734).

Variable	Categories	Whites adjusted HR (95% CI)^∗^	Blacks adjusted HR (95% CI)^∗^
Sex	Male/female	0.82 (0.77–0.88)	1.10 (0.84–1.44)

Age	Per year decrease	0.81 (0.79–0.83)	0.76 (0.68–0.83)

Stage	Localized	1.0 (ref.)	1.0 (ref.)
	Regional	1.26 (1.13–1.39)	0.98 (0.61–1.59)
	Distant	1.21 (1.11–1.31)	1.14 (0.77–1.67)
	Unknown	1.11 (0.98–1.25)	0.80 (0.44–1.45)

Cancer-directed surgery	No/yes	0.65 (0.61–0.70)	0.53 (0.38–0.73)

Radiation	No/yes	1.10 (1.01–1.20)	1.24 (0.81–1.89)

Therapy	None	1.0 (ref.)	1.0 (ref.)
	Surgery only	0.68 (0.64–0.73)	0.53 (0.37–0.74)
	Radiation only	1.27 (1.13–1.42)	1.24 (0.75–2.04)
	Both surgery and radiation	0.63 (0.55–0.71)	0.65 (0.30–1.39)

^∗^Adjusted for the other variables in the table.

**Table 3:** Receipt of surgery by stage among blacks and whites (*n* = 13734).

Summary stage	Whites		Blacks	
	Surgery performed		Surgery performed	
	No *N* (%)	Yes *N* (%)	No *N* (%)	Yes *N* (%)
Localized	992 (68.5)	457 (31.5)	53 (70.7)	22 (29.3)
Regional	1346 (62.4)	809 (37.5)	61 (67.0)	30 (33.0)
Distant	6002 (78.6)	1637 (21.4)	382 (84.9)	68 (15.1)
Unknown	1619 (89.8)	184 (10.2)	67 (93.0)	5 (7.0)
